# Perspective: on the importance of extensive, high-quality and reliable deposition of biomolecular NMR data in the age of artificial intelligence

**DOI:** 10.1007/s10858-024-00451-w

**Published:** 2024-10-20

**Authors:** Victoria A. Higman, Eliza Płoskoń, Gary S. Thompson, Geerten W. Vuister

**Affiliations:** 1 Department of Molecular and Cell Biology, Leicester Institute of Structural and Chemical Biology, University of Leicester, Leicester, LE1 7RH UK; 2 Wellcome Trust Biological NMR Facility, School of Biosciences, Division of Natural Sciences, University of Kent, Canterbury, Kent, CT2 7NZ UK

**Keywords:** NMR, Dynamics, Database, NMR exchange format, BMRB

## Abstract

Artificial intelligence (AI) models are revolutionising scientific data analysis but are reliant on large training data sets. While artificial training data can be used in the context of NMR processing and data analysis methods, relating NMR parameters back to protein sequence and structure requires experimental data. In this perspective we examine what the biological NMR community needs to do, in order to store and share its data better so that we can make effective use of AI methods to further our understanding of biological molecules. We argue, first, that the community should be depositing much more of its experimental data. In particular, we should be depositing more spectra and dynamics data. Second, the NMR data deposited needs to capture the full information content required to be able to use and validate it adequately. The NMR Exchange Format (NEF) was designed several years ago to do this. The widespread adoption of NEF combined with a new proposal for dynamics data specifications come at the right time for the community to expand its deposition of data. Third, we highlight the importance of expanding and safeguarding our experimental data repository, the Biological Magnetic Resonance Data Bank (BMRB), not only in the interests of NMR spectroscopists, but biological scientists more widely. With this article we invite others in the biological NMR community to champion increased (possibly mandatory) data deposition, to get involved in designing new NEF specifications, and to advocate on behalf of the BMRB within the wider scientific community.

Slightly over 50 years ago, in October 1971, the Protein Data Bank (PDB) was announced as a joint effort between the Cambridge Crystallographic Data Centre in the UK and the Brookhaven National Laboratory in the US (Letter 1971). The aim was to harvest and make available to researchers the experimental atomic co-ordinates, structure factors and electron density maps of protein structures solved by X-ray crystallography. These data represented a supplement to the publication of these structures in conventional scientific journals. In these early, pre-internet days, storage and distribution of the data was primarily done on magnetic tapes. As the initial announcement pointed out: “The success of the proposed system will depend on the response of the protein crystallographers supplying data” (Letter 1971). The response of the crystallographic scientific community proved to be very good and over the past five decades the PDB has grown and turned into the world-wide PDB (wwPDB) (Berman et al. 2003). It has added an additional distribution partner in Japan, the Biological Magnetic Resonance Data Bank (BMRB/NMRHub) for experimental NMR data, and most recently also a distribution hub in China (Xu et al. 2023). The wwPDB has also been accepting protein structures determined by other techniques such as Nuclear Magnetic Resonance (NMR) spectroscopy and Electron Microscopy (EM). These days, anyone wishing to publish an article describing a new protein structure is required by journals to deposit the structure and associated data with the wwPDB.

Fifty years after the launch of the PDB, advances in computational power allowed DeepMind to use the by then ~ 180,000 deposited structures to develop AlphaFold2, an artificial intelligence model capable of predicting many protein structures with remarkable accuracy from the primary sequence alone (Jumper et al. 2021). While it is tempting to assume that the “protein structure problem has now been solved”, AlphaFold2’s successes have not signalled the end of experimental structural biology (Terwilliger et al. 2023). On the contrary, over the past five decades our understanding of protein structure, dynamics and the structure-function relationship has shifted dramatically. We now understand that not all protein sequences will fold into stable 3-dimensional structures (Wright and Dyson 1999; Arai et al. 2024) and that the dynamics of a protein, whether local or global, is often integral to a protein’s function (Palmer 2004; Eisenmesser et al. 2005; Alderson and Kay 2021). NMR plays an exquisitely important role in characterising the dynamics of folded proteins across a variety of time scales (Palmer 2004), as well as providing a key experimental tool for studying the dynamics and residual structure of intrinsically disordered proteins (IDPs) (Milles et al. 2018; Ahmed and Forman-Kay 2022). Relaxation rates, paramagnetic relaxation enhancements (PREs), residual dipolar couplings (RDCs) and chemical shifts can all contribute to our understanding of protein dynamics and function (Palmer 2004; Milles et al. 2018; Schneider et al. 2019; Alderson and Kay 2021) including otherwise inaccessible excited state structures (Baldwin and Kay 2009).

In the same way that AlphaFold2 can predict the static structure of a protein from the primary sequence alone, might it one day also be possible to predict the dynamical behaviour of a protein from the primary sequence alone? It does not look like this will become a reality any time soon, most importantly because the data likely required to underpin such work are currently not accessible. When publishing NMR resonance assignments and/or structures, the deposition with the wwPDB and BMRB of chemical shift values, restraints used in the NMR structure calculation and atomic co-ordinates is mandatory. However, when publishing studies based on other types of NMR data, such as titrations, relaxation rates or PREs, deposition of the underlying data is unfortunately not mandatory. Sometimes data are appended as Supplementary Information, and more recently some researchers have begun using repositories such as Zenodo (https://zenodo.org/), an OpenScience data storage facility run by CERN, or BMRbig (https://bmrbig.bmrb.io/), hosted by the BMRB, to deposit all of the data associated with an NMR project, including the unprocessed spectra, processing parameters, software project files etc. However, even if such data can be used for individual studies, the problem with an undocumented and non-formalised way of sharing data is that these are not easily mined using automated protocols and may not even contain the required information, thus strongly hampering future large-scale, AlphaFold-style studies. Overall, the amount of deposited NMR data such as spectra, peak lists or dynamics data in the BMRB are very low (cf. Table 1). These limitations are already hampering wide-scale AI-based analysis of NMR data. ARTINA, a novel program to assign the resonances and determine the structure of globular proteins directly from solution NMR spectra contains an AI-based method for peak picking (Klukowski et al. 2018, 2022). The algorithm was trained on a mere 100 datasets of globular proteins studied by solution NMR, since these were all that was available to the authors. By comparison, the number of non-redundant structures (< 90% sequence similarity) solved by solution NMR is nearly 9,000. Furthermore, in a wwPDB-wide study of possible multi-state NMR protein structures, comparisons with dynamics data have been hampered by the low numbers of dynamics datasets deposited (Roland Riek, personal communication). Although simulated training data has been successfully used for several NMR-based AI-models (Beckwith et al. 2021; Shukla et al. 2023), this approach is only suitable for NMR data processing and analysis, not in relating NMR data and parameters sensitive to protein dynamics and function back to protein structure and sequence (this would require currently unfeasible quantum mechanical simulations of 10,000s of atoms across a ms-s timescale).

**Table 1:** Number of deposited entries in the BMRB containing a variety of different measurable NMR parameters

Parameter^a^	Number of Entries	NEF specification^b^
Assigned Chemical Shifts	15,669	✓
Spectra	247	
Peak Lists	957^c^	✓
Heteronuclear NOE	352	(✓)
R1^d^	356	(✓)
R2^d^	342	(✓)
R1rho^d^	19	(✓)
^1^H Exchange Rates	26	
^1^H Exchange Protection Factors	4	
Coupling Constants	384	
RDCs	152	✓
Chemical Shift Anisotropy	7	
Binding Data (K_d_)	73	
Structures^e^	14,004	-
Restraints^e^	11,462	✓

^a^ It is currently not possible to retrieve PREs or pseudo contact shifts (PCSs). Titration series can be deposited as multiple chemical shifts lists measured on different samples or samples with different conditions. However, they are not identified as titration series. Only 60 entries contain at least 5 chemical shift lists, but many of these are not titration series; thus, the number of deposited titration series is liable to be relatively small.

^b^ Parameters for which NEF specifications exist are indicated by a tick, those for which a proposal for a NEF specification has been made have a tick in brackets. Proposals for Coupling Constant, PRE and PCS NEF specifications have not been tabled yet.

^c^ It should be noted that a number of these entries do not contain NMR-STAR formatted peak tables but an NMR-STAR header with the peak list data in a comment in a different format (NMRPipe, NMRView, XEASY, Sparky etc.).

^d^ In the BMRB these are deposited as relaxation times, but for the new NEF specification we suggest using rates.

^e^ Structures and Restraints are deposited with the wwPDB rather than the BMRB.

A further problem for the deposition of NMR data has been the deposition format. When it was shown that the quality of NMR structures could be improved through the use of more modern structure calculation protocols (Nabuurs et al. 2004; Nederveen et al. 2005), a large-scale recalculation of structures was not found to be practicable without a large-scale remediation effort of deposited restraints involving much human intervention (Doreleijers et al. 2009). The critical assessment of automated structure determination of proteins by NMR (CASD-NMR) (Rosato et al. 2009, 2012, 2015) required extensive exchange of NMR data and validation of results. Again, this often required manual intervention, e.g. where header data was ambiguous or files were missing or damaged and for certain software packages stereo-specificity of restraints could not be taken into account (Ragan et al. 2015). Deposited peak lists also present a problem, since these are not curated by the BMRB and thus present in the BMRB in a variety of different formats and do not all conform to the BMRB’s NMR-STAR format. In 2015, following the second CASD-NMR round, nearly all key NMR software developers came together to develop what is now called the NMR Exchange Format (NEF; https://github.com/NMRExchangeFormat/NEF) in order to exchange NMR data seamlessly between software packages and deposit it with the BMRB (Gutmanas et al. 2015). NEF has been designed to be accurate in its documentation of data, e.g. stereo- and non-stereospecificity of assignments and restraints, is easily extendible, uses the STAR format (Hall 1991) already used by the wwPDB and BMRB, and is humanly readable. Crucially, we are tabling a proposal for documenting NMR dynamics data for consideration by the participants of the NEF effort (see https://github.com/NMRExchangeFormat/NEF/tree/master/specification) with a proposal for PRE and other data types to follow. The number of programs that are able to read and/or write NEF files now includes all the major structure calculation programs (Amber (Case et al. 2005, 2023), ARIA (Rieping et al. 2007), CS-Rosetta (Nerli and Sgourakis 2019), CYANA (Güntert and Buchner 2015), YASARA (YASARA Biosciences GmbH), XPLOR-NIH (Schwieters et al. 2003, 2006)), and several spectrum display software packages, e.g. CcpNmr Analysis (Skinner et al. 2016) and Sparky (Lee et al. 2015) as well as wwPDB/BMRB deposition. In addition, the program NEF-Pipelines (Thompson 2024) is enabling NEF-based access to numerous other programs that are not NEF compatible, as well as general manipulation of NEF files. The advent of NEF and its use by an increasingly large number of software packages should enable the easy deposition of a wide range of NMR data which correctly preserves the information content. To misquote the original PDB announcement: whether we do so, “will depend on the response of the NMR spectroscopists supplying data”. We are of the opinion that journal editors will likely have to mandate, rather than merely encourage deposition of all relevant data.

A separate format issue occurs in relation to binary (time and frequency domain) spectral data. These data are currently deposited in many different formats, depending on the spectrometer manufacturer and processing or spectral analysis software used by individual scientists. Although code libraries to read and write a variety of formats do exist (Helmus and Jaroniec 2013), a universal, simple, modern and well-documented format which is used for deposition would support both large-scale data mining and reproducibility and could become part of future efforts of the NEF Consortium.

Finally, a crucial hurdle is safeguarding the actual physical repository infrastructure, whose storage capacity will have to increase by several orders of magnitude if spectral data are to be deposited. This increase in capacity is particularly important considering that many modern spectra are recorded with non-uniform sampling which typically leads to much larger processed spectrum files. Curation of the data is essential, but expensive. The BMRB, as the wwPDB partner responsible for NMR data, is a US-funded project whose existence has too often appeared to be more precarious than it should be, given its importance to the global NMR community. It is imperative that not only the NMR community, but also the wider biological and scientific community acknowledges the importance of large repositories of scientific data in general and, therefore, also supports funding for the BMRB as securely as the wwPDB and other scientific repositories, possibly also through international efforts to provide greater robustness.

It should go without saying that the deposited data needs to be accurate, requiring good training and best practice to be in place across the community. The analysis of dynamics data, in particular, is not straight forward and robust validation methods need to be developed alongside the analysis methods themselves. We are currently developing tools to enable this within the CcpNmr AnalysisAssign program (Skinner et al. 2016). A further advantage to depositing NMR datasets and measured data is that it opens up the possibility of reanalysing the deposited data with these and other improved methods as they become available.

While experimental structure determination may continue to be our gold-standard for the study of protein structures, programs such as AlphaFold2 represent a highly useful tool for biologists studying proteins which have so far eluded experimental structure determination. Given the importance of protein dynamics and IDP residual structure for protein function it would be highly desirable to have similar tools available for their prediction in the absence of detailed experimental data. If AlphaFold2 has taught us anything, it is the value of large, well-organised data repositories such as the wwPDB. Modern artificial intelligence systems are capable of detecting patterns in large datasets which humans are not, even with the help of the best traditional algorithms. If we want to be able to make the most of this new technology, we need to ensure that all of our experimental data are deposited in a high-quality, reliable way, starting as soon as possible (Editorial 2023; Arrowsmith 2024). We therefore invite the community to participate in the discussion about the expansion of the NEF specifications for data exchange and deposition, as well as ways to increase or mandate data deposition and safeguard our repositories for the future. This will be good not only for NMR spectroscopy as an experimental technique to study proteins and other biological molecules, but for biology and science, in general.

## Data Availability

No datasets were generated or analysed during the current study.
